# Initial clinical experience of non-invasive treatment of Magnetic Resonance guided high intensity focused Ultrasound (MRgFUS) for focal breast cancer

**DOI:** 10.1186/2050-5736-2-S1-A16

**Published:** 2014-12-10

**Authors:** Beatrice Cavallo Marincola, Alessandro Napoli, Federica Pediconi, Luisa Di Mare, Carola Palla, Marianna Telesca, Elena Miglio, Maria Ida Amabile, Grazia Marangi, Giulia d’Amati, Massimo Monti, Carlo Catalano

**Affiliations:** 1 Sapienza University of Rome – Department of Radiological, Oncological and Anatomopathological Sciences, Rome, Italy; 2 Sapienza University of Rome - Department of Surgical Science, Rome, Italy

## Background

Breast cancer is the first cause of oncologic female death in western countries [1]. Current medicine is continuously looking for a treatment that is the least invasive and the maximum conservative as possible.

Magnetic Resonance guided Focused Ultrasound (MRgFUS) is a non-invasive ablation technique that relies on the use of a high-intensity focused ultrasound beam that can increase the punctual temperature within a target tissue, causing a consequent thermal damage and coagulation necrosis [2,3]. The Magnetic Resonance guidance allows a better visualization of the lump and the real time temperature monitoring during the entire treatment [4].

## Materials and Method

The aim of this multicentric, prospective study is to assess safety and feasibility of MRgFUS ablation of invasive ductal breast cancer (IDC) (stage T1 N0 M0).

Main inclusion criteria are: women > 18 years-old with biopsy-proven invasive ductal carcinoma (T1 N0 M0); the presence of a single lump with 2 cm maximum diameter visible at contrast-enhanced MRI; a minimum distance of 10 mm between the lump and the skin layer, the pectoral muscle and the nipple.

All procedures were performed on 3T MR scanner (GE Medical Systems), featuring a HIFU transducer embedded into the patient table (ExAblate 2100, InSightec, Haifa - Israel). Treatment was performed under conscious sedation with patients placed in prone position. Baseline and gadolinium-enhanced sequences were acquired in order to define the ablation area and confirm the correct patient positioning.

Ablation volume included the enhancing tumor + 5 mm margin. Skin layer and rib cage were defined in order to be protected from high energy levels. The system automatically generated a treatment plan taking into account the anticipated energy delivered through the skin to the region of treatment. After verifying the correct target area using low-power sub-therapeutic sonications, treatment started at full therapeutic power. At the end of each treatment, the same sequences performed before the procedure were acquired to confirm the ablation area and the absence of thermal damage to adjacent tissues. Patients were examined for any adverse events and monitored for 3-4 hours prior to discharge. Within 14 days after the procedure all the patients underwent surgical lumpectomy for the histopathology analysis.

## Results

**Figure 1:**
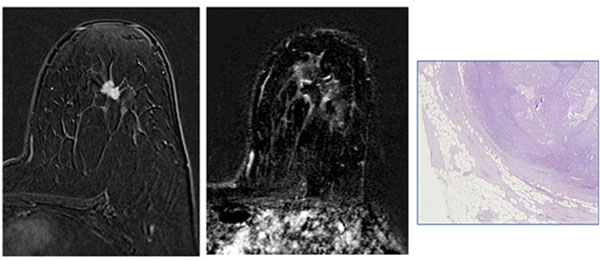
47-years old female with left breast IDC. Contrast enhanced fat suppressed 3D T1-weighted GRE sequence acquired in axial plane (a) shows a 10 mm lump located between upper quadrants (arrows). After MRgFUS treatment the same sequence (b) shows the complete absence of enhancing tissue surrounded by some hyperemia due to gland inflammation. Microscopy (c) confirmed the necrosis area surrounded by fibrotic tissue.

Five patients have been treated in the study. In 3/5 patients histopathology confirm 100% necrosis of the tumor surrounded by fibrosis (Fig. 1). In the remaining 2 patients histopathology found respectively 15% and 70% of residual tumor; in this latter case in particular, an irreversible damage of the transducer was found, probably due to reflection of the ultrasound beam caused by possible air bubbles between the transducer and the skin.

No treatment-related adverse events were recorded after the treatment or during the follow-up period prior to lumpectomy (10-15 days).

## Conclusion

Even if the study is still in progress, our preliminary results show that MRgFUS is a safe and effective non-invasive ablation modality in patients with localized breast invasive ductal cancer.
